# Inverse Relationship between Tumor Proliferation Markers and Connexin Expression in a Malignant Cardiac Tumor Originating from Mesenchymal Stem Cell Engineered Tissue in a Rat *in vivo* Model

**DOI:** 10.3389/fphar.2013.00042

**Published:** 2013-04-17

**Authors:** Cathleen Spath, Franziska Schlegel, Sergey Leontyev, Friedrich-Wilhelm Mohr, Stefan Dhein

**Affiliations:** ^1^Translational Centre for Regenerative MedicineLeipzig, Germany; ^2^Clinic for Cardiac Surgery, Heart Centre LeipzigLeipzig, Germany

**Keywords:** connexins, gap junctions, sarcoma, proliferation, tumour, Cx43, Cx40, Cx45

## Abstract

**Background:** Recently, we demonstrated the beneficial effects of engineered heart tissues for the treatment of dilated cardiomyopathy in rats. For further development of this technique we started to produce engineered tissue (ET) from mesenchymal stem cells. Interestingly, we observed a malignant tumor invading the heart with an inverse relationship between proliferation markers and connexin expression.

**Methods:** Commercial CD54^+^/CD90^+^/CD34^−^/CD45^−^ bone marrow derived mesenchymal rat stem cells (cBM-MSC), characterized were used for production of mesenchymal stem-cell-ET (MSC-ET) by suspending them in a collagen I, matrigel-mixture and cultivating for 14 days with electrical stimulation. Three MSC-ET were implanted around the beating heart of adult rats for days. Another three MSC-ET were produced from freshly isolated rat bone marrow derived stem cells (sBM-MSC).

**Results:** Three weeks after implantation of the MSC-ETs the hearts were surgically excised. While in 5/6 cases the ET was clearly distinguishable and was found as a ring containing mostly connective tissue around the heart, in 1/6 the heart was completely surrounded by a huge, undifferentiated, pleomorphic tumor originating from the cMSC-ET (cBM-MSC), classified as a high grade malignant sarcoma. Quantitatively we found a clear inverse relationship between cardiac connexin expression (Cx43, Cx40, or Cx45) and increased Ki-67 expression (Cx43: *p* < 0.0001, Cx45: *p* < 0.03, Cx40: *p* < 0.014). At the tumor-heart border there were significantly more Ki-67 positive cells (*p* = 0.001), and only 2% Cx45 and Ki-67-expressing cells, while the other connexins were nearly completely absent (*p* < 0.0001).

**Conclusion and Hypothesis:** These observations strongly suggest the hypothesis, that invasive tumor growth is accompanied by reduction in connexins. This implicates that gap junction communication between tumor and normal tissue is reduced or absent, which could mean that growth and differentiation signals can not be exchanged.

## Introduction

There is a long standing debate about factors that may be involved in the invasive growth of tumors. Among these gap junctions have been discussed since Werner Loewenstein proposed that gap junction intercellular communication (GJIC) might play a role in tumorigenesis and that reduced communication may account for loss of growth inhibition (Loewenstein and Kanno, 1966; Loewenstein, 1980). Since that time a number of papers has shown that in primary tumors or tumor cell lines connexins can be downregulated or even be absent, that oncogenes or cancerogenic drugs often inhibit gap junction channel function or reduce connexin expression (Loewenstein and Kanno, 1966; Trosko et al., 1990; Lampe, 1994; Laird et al., 1999; Mesnil et al., 2005; Salameh and Dhein, 2005; Cronier et al., 2009). However, on the other hand, some researchers found a role of gap junctions promoting invasion, cell extravasation, and migration of tumor cells (Naoi et al., 2007; Saito-Katsuragi et al., 2007; Ezumi et al., 2008), while others do not support this view (Yano et al., 2006; Sato et al., 2008). This lead to the interpretation that connexins might be “differentially regulated during the dissemination of specific tumor types” (Naus and Laird, 2010), and that down-regulation of connexin in early tumors might be linked to invasion, but in later states elevation in connexins can occur facilitating extravasation and formation of secondary tumors (Naus and Laird, 2010). Regarding tumor cells, GJIC may exist among normal or (pre-)cancerous cells (homologous GJIC) or between normal and (pre)cancerous cells (heterologous GJIC), (Yamasaki et al., 1995), or may be absent. Thus, an open question is, whether there are gap junctions at the border between a tumor and the neighboring tissue. Another issue of debate is the role of connexins in stem cells (Trosko et al., 2004). In recent years, mesenchymal stem cells (MSC) gained huge interest in regenerative medicine and tissue engineering. To their exceptional advantages belongs their multipotent differentiation capacity to cell lineages, including myocytes, osteocytes, chondrocytes, tenocytes, and adipocytes (Pittenger et al., 1999). Additionally, MSC can be derived from different tissues, like bone marrow (BM), adipose tissue, and umbilical cord (Zuk et al., 2001; Romanov et al., 2003). Until today, MSC, mostly BM-MSC, have been successfully transplanted in animal models as well as in human patients suffering from for instance myocardial infarction, dilated cardiomyopathy, stroke, neuroimmunological, and neurodegenerative diseases (Horwitz et al., 1999; Bang et al., 2005; Nagaya et al., 2005; Karussis et al., 2008; Tang et al., 2009; Chin et al., 2010). Furthermore MSC have kept entering tumor research. Several *in vivo* and *in vitro* studies have shown the ability of MSC to inhibit tumor growth in different malignancies (Maestroni et al., 1999; Ohlsson et al., 2003; Nakamura et al., 2004; Khakoo et al., 2006; Tian et al., 2010). In contrast, various scientific groups observed, that MSC could promote metastasis (Karnoub et al., 2007) and enhance tumor growth (Gunn et al., 2006; Zhu et al., 2006; Spaeth et al., 2009), which is assumed to be attributable to for instance immunosuppression (Djouad et al., 2003) or drug resistance (Kurtova et al., 2009). Moreover several types of MSC may transform to malignant cells *in vitro* and *in vivo* (Rubio et al., 2005; Miura et al., 2006; Zhou et al., 2006; Tolar et al., 2007). Due to these contradictory observations, Wong (2011) discussed in her paper the question, whether MSC are “angels or demons.”

A kicking point about the role of a cell within a given tissue is the question, whether this cell can communicate with its neighboring cells, which may regulate growth and differentiation of the cell *via* gap junctional intercellular communication (Loewenstein, 1980; Trosko et al., 1990). In that context, an even older standing debate than the scientific efforts in mesenchymal stem cells is the questions about the role of connexins in tumor growth and communicating with its surrounding. Gap Junction channels are made from two hemichannels (connexons) contributed by either of the neighboring cells. A connexon consists of 6 connexins, 4-transmembrane spanning proteins, with an intracellular N- and C-terminal. Twenty-one connexin isoforms are presently known, which – besides other properties – differ in their molecular weight, their gating properties, and their tissue distribution (Evans and Martin, 2002; Söhl and Willecke, 2004).

Another open question in current regenerative medicine, in particular cardiovascular approaches to BM-MSC therapy by BM-MSC injection, is whether adult BM-MSC can form malignant tumors or not, and whether in such a case these cells may communicate with normal tissue. In favor of this idea Valiunas et al. (2004) showed in commercially available human MSC that these cells can express Cx43, Cx40, and Cx45, and found punctuate Cx43 and Cx40 staining in regions of close cell–cell contact, while Cx45 was mostly found cytoplasmically. In addition, they showed that these cells formed functional gap junction channels within their population and with transfected HeLa cells.

In an investigation, which originally was aimed to investigate the possible use of BM-MSC for cardiac tissue replacement therapy by using these cells to form engineered heart tissue instead of neonatal rat cardiomyocytes, which have been previously used (Zimmermann et al., 2006; Leontyev et al., 2013) we observed tumor formation in mesenchymal stem-cell-engineered tissue (MSC-ET) after transplantation *in vivo*, and found characteristic gap junction protein distributions.

The primary idea behind our study was to replace cardiomyocytes in engineered heart tissue (Leontyev et al., 2013) by mesenchymal stem cells. To our surprise we observed a malignant tumor originating from the ET. This tumor showed an interesting reverse relationship between proliferation and expression of the cardiac connexins Cx43, Cx40, and Cx45. Thus, those areas where the tumor invaded the heart, were negative for cardiac connexins but positive for Ki-67. In contrast – areas in the middle of the tumor were negative for the proliferation marker Ki-67 but positive for connexins. Thus, this tumor exhibits both Cx-positive and -negative areas.

## Materials and Methods

### Used MSC lines

We used two types of BM-MSC: (a) isolated by ourselves (sBM-MSC; see below) and (b) a commercial rat BM-MSC (cBM-MSC) from Gibco (S1601-100; Gibco, Darmstadt, Germany).

### BM-MSC isolation

For the isolation of sBM-MSC we used male Sprague Dawley rats weighing about 250–350 g. The rats were anesthetized with: fentanyl (0.005 mg/kg), midazolamhydrochloride (2 mg/kg), medetomidinehydrochloride (0.15 mg/kg), and ketamine (75 mg/kg). Afterward the rats were killed by excising the heart. The femora and tibias were dissected aseptically and washed with PBS. The bone epiphyses were cut off and each remaining diaphysis was placed in one pipette tip, which then was put in a Falcon tube. In order to get the bone marrow out of the cavities we centrifuged the bones (200 × *g*, 5 min, 21°C) (Dobson et al., 1999). The cell pellets were purified from tissue remnants by a 100 μm^2^-filter and seeded on 75 mm^2^-flasks (one pellet per flask).

### MSC culture

Both cell lines were cultured at 5% CO_2_ and 37°C in Dulbecco modified Eagle medium-low glucose supplemented with 10% fetal bovine serum and 2.5% Streptomycin/Penicillin. The first medium change was performed after 3 days to remove the non-adherent cells (Strawn et al., 2004). Afterward the medium was removed every 3–4 days. When the MSC reached 80% confluence, they were trypsinized and plated at a density between 2 and 6 × 10^3^/cm^2^.

### Characterization of MSC: Flow cytometry and adipogenic differentiation

For characterization we performed flow cytometry and adipogenic differentiation for three cultures of sBM-MSC (passage 3). For cBM-MSC (passage 6) we could validate the manufacturer’s descriptions (CD29+, CD44+, CD90+, CD106+ (>70%), CD11b−, CD34−, CD45− (<5%) and adipogenic-, chondrogenic-, and osteogenic-differentiation).

Using flow cytometry analysis (LSR II, Becton Dickinson, Heidelberg, Germany) we analyzed expression of two MSC-markers: CD54 and CD90 and two negative-markers: CD34 and CD45 (Pittenger et al., 1999; Wan et al., 2008; Hong et al., 2009). Briefly, cells were incubated with FITC-mouse monoclonal antibodies against rat – CD34 (Santa Cruz; sc-7324), CD45 (BD; 550616), CD54 (BD; 554969), and CD90 Thy1/Thy1.1 (BD; 554894). For the control served isotype-identical antibodies: Mouse IgG1 κ (BD; 550616) and Mouse IgG2a κ (BD; 553456). Labeled cells were detected on a LSR II (Becton Dickinson, Heidelberg, Germany) by collecting 10.000 events using FACSDiva software (Becton Dickinson, Heidelberg, Germany).

To proof evidence of multipotency of MSC we induced adipogenic differentiation by the STEMPRO^®^ Adipogenesis Differentiation Kit (Gibco, Darmstadt, Germany). After 3 weeks, lipid vacuoles within cells were detected by Oil-Red-O-staining in both cell lines (Pittenger et al., 1999).

### Engineered tissue production from MSC (MSC-ET production)

For creating a MSC-ET, 2.5 × 10^6^ cultured MSC/ml, either (a) sBM-MSC or (b) cBM-MSC, were mixed with matrigel, collagen I, and serum containing media and were formed to rings by casting into a circular structure as described (Leontyev et al., 2013). Briefly, for preparing ET rings, 5 × 10^6^ cells suspended in a total volume of 910 μl M199 with 20% fetal calf serum and penicillin/streptomycin were mixed 200 μl matrigel (Becton Dickinson, Heidelberg, Germany), 500 μl collagen I (5.5 mg/ml; Sigma, Taufkirchen, Germany), 70 μl 0.1 M NaOH (4%) and 320 double concentrated M199 medium containing 20% horse serum and 4% fetal calf serum, the pH was adjusted to 7.4 with HEPES (Zimmermann et al., 2006). The cell mixture was cast into a circular ring-like structure of 10 mm diameter and 2 mm wall thickness and allowed to solidify for 1 h. Thereafter, M199 media was added carefully. After 24 h medium was changed. ET rings were then allowed to grow in M199 media (supplemented with 10% horse serum, 1% fetal calf serum, 2% chick embryo extract). After 5 days of consolidation time MSC-ETs were electrically stimulated (1 Hz, 1 mV, and 0.1 mA) for further 6 days. In total the MSC-ETs were cultivated for 2 weeks. We obtained six mesenchymal stem cell Engineered Tissues (sMSC-ET) from sBM-MSC (passage 3) and seven cMSC-ETs from cBM-MSC (passage 6).

### Chronic MSC-ET implantation in the rat

Subsequently three of six sMSC-ETs and three of seven cMSC-ETs were implanted around the beating heart of adult female Sprague Dawley rats, which were obtained from University of Leipzig (the remaining BM-ETs were used for pre-implantation histology, see below). The ring-like MSC-ETs were implanted around the whole heart, i.e., around the free wall of the left and right ventricles in a circular manner below the valve plane. Rats were housed in controlled facility with 12:12 h light/dark cycle with standard laboratory diet.

In animals undergoing surgery: rats were anesthetized by combined intramuscular application of fentanyl (0.005 mg/kg), midazolam (2 mg/kg); medetomidin (0.15 mg/kg) and after intubation isoflurane (1.5%), which was continued as inhalation anesthesia (0.8–1% isoflurane). The thorax was opened by sternotomy. The MSC-ET was directly implanted from the culture disk to the animal. MSC-ET rings were placed on the beating heart in the middle of the left and right ventricle and actively fixed with four to five (8/0 Prolene, Eticon) single-knot sutures.

All animals received immunosuppression for 30 days (day 80–110) with cyclosporin (5 mg/kg/day), methylprednisolone (5 mg/kg/day), and azathioprine (2 mg/kg/day). After 1 month the rat hearts were surgically excised and preserved for histological analysis of the MSC-ETs *in vivo*.

### Histology

Cultivated *in vitro* MSC-ETs and rat hearts with the transplanted MSC-ETs were fixed in 4% buffered formaldehyde solution and embedded in paraffin for cutting 4 μm thick sections, and processed for standard Hematoxylin Eosin staining, and Azan-novum, Elastica van Gieson and immunohistological staining. For evaluating the amount of collagen fibers of the MSC-ET we used the Azan-novum staining. Therefore the object slides were incubated for 7 min in 0.1% Kernechtrubin solution, which stained nuclei red. Subsequently collagen tissue was stained blue by 5 min in aniline blue-Orange G. Existence of elastic fibers could be shown by Elastica-van-Gieson-staining. Paraffin slides were put in resorcin-fuchsin for 15 min and elastic fibers turned into a dark purple. Nucleoli were stained brown by incubating for 5 min in Weigert’s Hematoxylin.

### Immunohistochemistry

After a 10 min rinsing step in TBS, 4 μm paraffin sections were immersed in 0.01 M sodium citrate buffer (pH 6.0) and cooked in a microwave for 30 min at 600 W. The endogenous peroxidase activity was inhibited by incubating for 10 min in a solution consisting of 60% methanol, 40% TBS, and 0,3% hydrogen peroxide. Microscopic slides were blocked with 2% BSA for 1 h and afterward they were incubated with 1:100 diluted rabbit polyclonal anti-von Willebrand Factor (Ab6994, Abcam, Cambridge, UK) overnight at 4°C. Subsequently, the samples were incubated with a secondary anti-rabbit peroxidize-conjugated antibody (1:200) for 1 h at room temperature. Von Willebrand Factor positive vessels were visualized by staining them red with the chromogen AEC for 20 min at room temperature and nuclei were counterstained by Mayer’s hematoxylin.

For Cx40, Cx43, Cx45, CD90, CD20, CD3, CD45, and ki-67 we used a polyclonal Cx40 antibody (AB1726, Millipore, Schwalbach, Germany), a polyclonal rabbit anti-rat Cx43 antibody (C6219, Sigma, Taufkirchen, Germany), a rabbit anti-Cx45 antibody (AB1742, Chemicon, Temecula, CA, USA), a mouse monoclonal CD90 antibody (ab225, Abcam, Cambridge, UK), a goat polyclonal anti-CD20 antibody (Sc-7735, Santa Cruz, CA, USA), a mouse monoclonal anti-CD45 antibody (Sc-53047, Santa Cruz, CA, USA), a goat polyclonal anti-CD3-ε antibody (Sc-1127, Santa Cruz, CA, USA), and a goat antibody against ki-67 (Sc7846, SantaCruz, CA, USA). The immunofluorescence protocols varied with regard to permeabilization and blocking. For each staining protocol exposure times and antibody dilutions were tested and optimized separately prior to the final experiments. The specificity of immunostaining for Cx40, Cx43, Cx45 was tested prior to these experiments using transfected HeLa cells as described (Hagen et al., 2009).

For Cx40 staining, the paraffin-fixed sections were permeabilized for 15 min in a TBS solution with 0.1% Trypsin and 0.1% CaCl_2_ (pH 7.8). For Cx43 labeling slides were put in sodium citrate buffer (pH 6.0) and cooked in a microwave for 30 min at 600 W. The Cx45 and CD90 slides were permeabilized for 30 min in 1% Triton in PBS and blocked for further 60 min in 2% BSA in PBS. Cx40 and Cx43 slides were blocked for 60 min in 2% BSA in TBS. Afterward the slides were incubated with anti-Cx40 (1:100), anti-Cx43 (1:2000), anti-Cx45 (1:100), anti-CD90 (1:100), or anti-ki-67 (1:100) overnight at 4°C. Next, the slides were incubated with the appropriate Alexa Flour 488 or Alexa Flour 555 conjugated secondary antibodies (1:250) (Sigma, Taufkirchen, Germany) at room temperature for 1 h. Background autofluorescence was inhibited immersing for 1 min in 0.1% Chicago Blue in TBS. Nuclei were stained for 1 min with DAPI in TBS.

For histomorphometric investigations single slides were taken and the total field area for 10 randomly selected fields was examined. The heart sections were analyzed by blinded observers by using microscopy Zeiss Axioplan2 Microscope (Jena, Germany) equipped with a digital microscope camera (AxioCam MRC5 Zeiss, Jena, Germany). Exposure times and intensity for taking immunofluorescence pictures were adjusted for the series of pictures. The digital images were analyzed using AxioVision 4.6 (Zeiss, Jena, Germany).

### Statistics

All data are given as MEANS ± SEM of *n* experiments. The data were statistically analyzed by ANOVA, and if ANOVA indicated significance, by *post hoc* Tukey HSD or by a Chi-square test as appropriate on a level of significance of 0.05 using SYSTAT software (SYSTAT 11.0; Jandel Scientific, Erkrath, Germany).

## Results

### FACS analysis and adipogenic differentiation of adult mesenchymal stem cells prior to tissue engineering and transplantation

Bone marrow derived mesenchymal stem cells (passage 3) exhibited an expression of the mesenchymal surface markers CD90 and CD54, and to a lower extent for the hematopoietic marker CD45, while they were negative for the second hematopoietic marker CD34. In comparison the commercial cBM-MSC (passage 6) also were positive for CD90 and CD54, but were negative for both CD34 and CD45 (Table 1).

**Table 1:** **characteristics of the two groups of bone marrow derived stem cells used in this study**.

%	sBM-MSC	cBM-MSC
CD90	94 ± 3	99 ± 1
CD54	72 ± 4	96 ± 1
CD34	0 ± 0	0 ± 0
CD45	13 ± 2	0 ± 0
Adipocyte formation rate	10 ± 1	22 ± 0.3

*The percentage of cells which was positive for a certain antigen (FACS analysis; mesenchymal marker: CD90, CD54; hematopoietic markers: CD34, CD45) is given as well as the percentage of MSC which could be transformed to adipocytes. All values are given as means ± SEM*.

Both types of BM-MSC showed the ability of adipogenic differentiation, which was somewhat higher in cBM-MSC. After 3 weeks of incubation with adipogenic supplementation, 10 ± 0.93% of the sBM-MSC and 21.69 ± 0.31% of the cBM-MSC developed Oil-Red-O-positive lipid droplets (Table 1). Non-treated control cultures did not show spontaneous adipogenic formations after 3 weeks of cultivation (not shown).

### Formation of MSC-ETs *in vitro* and *in vivo*

**Figure 1:**
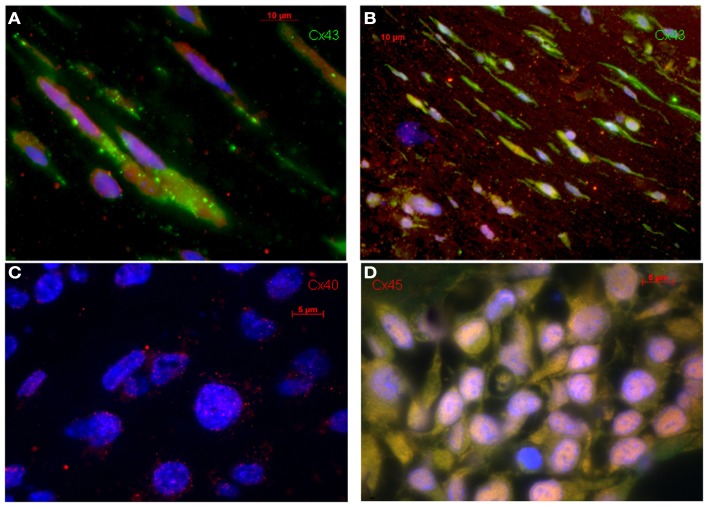
**Original immunohistological images from *in vitro* MSC-ETs**. **(A)** sMSC-ET and **(B)** cMSC-ET. Red: troponin I, green: Cx43, blue: DAPI. In both types no cross striation for troponin I can be seen. There are numerous cells expressing Cx43. However, Cx43 is mostly located intracellularly and is distributed irregularly. **(C,D)** Show Cx40 and Cx45 staining in cMSC-ET. Note the slight punctuate Cx40 staining [red; **(C)**] and the absence of a specific (red) Cx45 staining **(D)**.

The MSC-ETs (sMSC-ETs; cMSC-ETs) did not exhibit any contractions. Histological analysis of *in vitro* MSC-ET after 14 days of culture showed that both types of MSC-ET had a high amount of collagen, no elastic fibers, only very few vessels, almost no myocytes (defined as troponin I expressing cells), but considerable numbers of Cx43 or CD90 positive cells (see Table 2; Figure 1). Under *in vitro* conditions the MSC-ETs did not exhibit specific Cx45 but showed slight Cx40 staining (Figures 1C,D). After 30 days of implantation, the collagen content and the percentage of cells positive for the mesenchymal marker CD90 was reduced, while the number of vessels was increased, and a new formation of elastic fibers could be observed (Table 2).

**Table 2:** **Characteristics of the engineered tissues (ET) made from the two groups of bone marrow derived stem cells used in this study**.

%	*In vitro* ET		*In vivo* ET	
	sMSC-ET	cMSC-ET	sMSC-ET	cMSC-ET
Troponin^+^ cells	0.3 ± 0.1	0.3 ± 0.2	0.5 ± 0.2	0.1 ± 0.05
Cx43^+^ cells	38 ± 2	74 ± 4#	57 ± 9*	50 ± 11*
CD90^+^ cells	38 ± 14	77 ± 5#	16 ± 4*	16 ± 7*
Vessels/mm^2^	4 ± 1	20 ± 8#	49 ± 9*	39 ± 2*#
Collagen	70 ± 2	64 ± 2	37 ± 3*	28 ± 9*
Elastic fibers	0	0	1.8 ± 0.2*	0.9 ± 0.15*

*The percentage of cells which was positive for a certain antigen (CD90, Cx43, troponin I) is given as well as the number of vessels/mm^2^ and the percentage of the area which was covered by collagen or elastic fibers. All values are given as means ± SEM. Significance (*in vivo* vs. *in vitro* are indicated by an asterisk, significance between sBM-ET and cBM-ET is marked by # (*p* < 0.05)*.

Surprisingly, in one of the three cMSC-ET-transplanted rats the heart was completely surrounded by a sarcoma originating from the cMSC-ET, which nearly filled the whole thorax. This was not observed with sMSC-ETs. This tumor showed highly interesting features regarding the distribution and expression of gap junction proteins showing no intercellular gap junction protein expression at the border between heart and tumor, while the tumor expressed connexins in its inner center (but see below). Such tumor formation was not observed in any of the other rats.

### Morphology and macroscopic anatomy of the sarcoma

**Figure 2:**
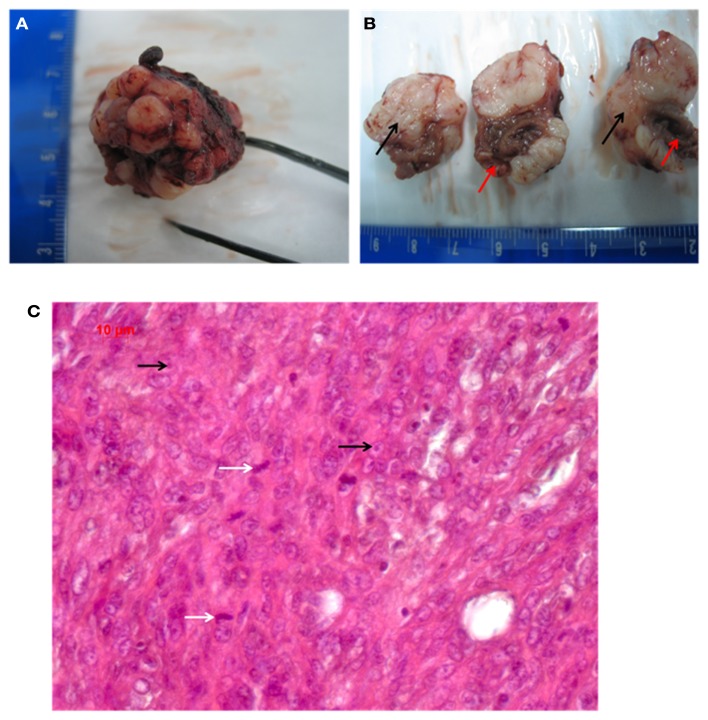
**(A,B)** Macroscopic pathology of the tumor (red arrows point to the heart, black arrows indicate the grayish tumor mass). **(C)** Shows the HE histology (white arrows indicate mitosis; black arrows point to nucleoli).

The measured diameter of the solid tumor was from minimum 3.28 mm to maximum 12.39 mm. In comparison, the heart exhibited a size of 9.03 mm (diameter measured at the widest transverse section) and 13.48 mm (at the longitudinal section). The solid neoplasm was well bounded and of tubercular shape. The intersection was white-greyly colored and it showed various lesions as indicated by bleedings and necrosis, which reminded to the typical description of a sarcoma (Fletcher, 1992). Figures 2A,B show the macroscopic aspect of this tumor.

### Pathology

Regarding the growth and location of the tumor, it became obvious that the tumor was originating from the cMSC-ET. The ET had been implanted around the rat heart and the growth direction of the neoplasm was from the ET to the heart with several infiltrations of the cardiac tissue.

The picture of the HE-staining (Figure 2C) was characterized by closely, side by side laying cells and sparsely surrounding collagen. The cells exhibited atypical nuclei and atypical cytoplasm. The pleomorphic cells showed different sizes and their shape was from clumsily oval through to round and only a few spindle shaped. Some nuclei were equipped with nucleoli, but an eminent majority of them were oversized and had a vesicular appearance, which resulted in hypochromatic staining. The type of growth differed between undirected and storiform and a few septums made of collagen tissue caused a knotty division. Many inflammatory infiltrates, predominantly consisting of lymphocytes, were found at the transition between the neoplasm and the heart, in particular, at spots, where the tumor had infiltrated the cardiac tissue. Atypical, polynuclear giant cells were not detected, as would be expected for a malignant giant cell tumor of soft tissues (Guccion and Enzinger, 1972; Angervall et al., 1981). By HE-staining we did not find any differentiation characteristics such as lipoblasts or osteoid formation, together with a high nucleus/plasma ratio, which implied, that the tumor is probably an undifferentiated pleomorphic sarcoma. A lymphoma could be excluded by the finding that the tumor cells were negative for CD3, CD20, and CD45.

### Grading

Following the hypothesis of this tumor being a sarcoma, there are two acknowledged grading systems for soft tissue sarcomas. We used the FNCLCC-system (Fédération Nationale des Centre de Lutte contre le cancer), because it correlates better with the clinical prognosis than the NCI-System (National Cancer Institute) (Guillou et al., 1997). The FNCLCC-system is based on three parameters: tumor differentiation, mitotic activity and necrosis, whereas the grading depends crucial on the histological type. According to the FNCLCC-system the tumor in this case exhibited an undifferentiated pattern, which couldn’t clearly dedicated to one special type (=score 3). Moreover, we found 41 mitoses in 10 high-power fields (1 HPF = 0.1734 mm^2^), which also gives a score value of 3. Less than 50% of the tumor tissue consisted of necrotic areas (=score 1), so that the total score was 7 (=7/8) corresponding to a high grade malignant sarcoma (Guillou et al., 1997; Deyrup and Weiss, 2006).

### Distribution of the connexins 40, 43, and 45 in the sarcoma and the heart tissue

**Figure 3:**
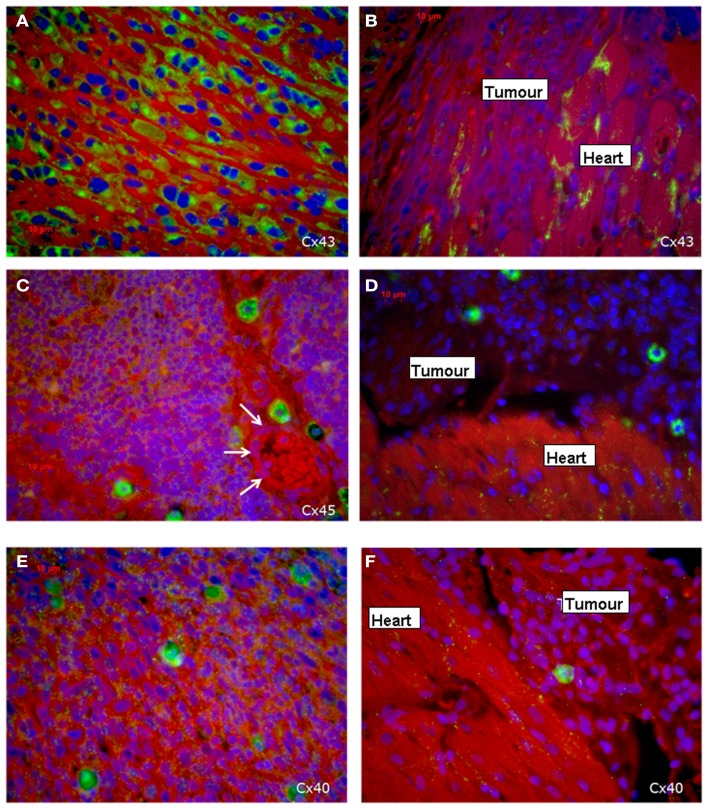
**Expression of the three cardiac connexins Cx43 (A,B), Cx45 (C,D), and Cx40 (E,F) (connexins are stained green)**. **(A,C,E)** Show the expression within the middle of the tumor, while **(B,D,F)** demonstrate the loss of connexin expression in the invading zone of the tumor close to the heart and a regular distribution of connexins in the heart itself. The white arrows indicate a vessel.

Immunohistochemical investigations revealed that the tumor cells in the middle of the tumor were positive for all three cardiac connexins, i.e., connexin 40, 43, and 45. By comparing the fluorescence signals of the three connexins, we recognized that most cells stained positive for Cx43, while a smaller percentage expressed Cx45 and Cx40. All of the three connexins were uniformly located in the cytoplasm of the sarcoma cells, while the plasma membrane was almost free of an immunopositive connexin signal and there was no accentuation of the connexins near the nucleus. In particular, there were no selective accumulations of the fluorescence between adjacent cells at the cell–cell-borders, which implies that functional gap junctions are less probable (Figure 3). In contrast, in the heart we found the typical localization of connexins at the plasma membrane with accentuation at the cell–cell contacts in particular at the cellular poles (Figures 3B,D,F).

Regarding the connexin expression, the sarcoma cells in the present tumor can be divided into two areas: the first is the sarcoma itself and the second is the transition zone between cardiac tissue and sarcoma including the infiltrative growing sarcoma cells (Figure 3B). The vast majority of sarcoma cells in the middle and outer area of the tumor are positive for the three connexins (Figures 3A,C,E), while in close vicinity of the transition zone the sarcoma cells lost their positive connexin signal (Cx43, Cx40, Cx45), which indicated that the tumor cells at the border do not express any of the cardiac connexins (Figures 3B,D,F). In the heart Cx43 is most prominently expressed.

**Figure 4:**
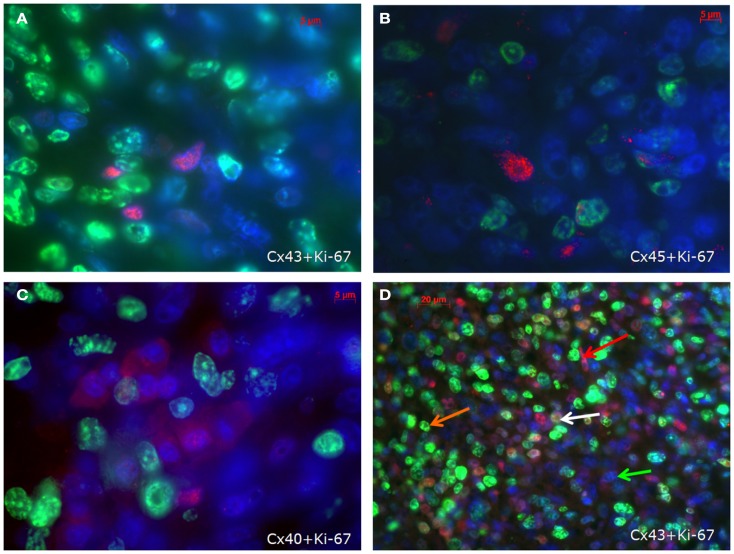
**Co-staining of Ki-67 (green) and Cx43 (A), Cx45 (B) and Cx40 (C) (connexins are stained red) in the inner part of the tumour**. In (**D**) an overview is shown at a lower magnification. Besides cells negative for both antigens (green arrow), cells are either positive for Ki-67 (orange arrow) or for Cx43 (red arrow). Only few cells co-express Cx43 and Ki-67 (white arrow).

**Figure 5:**
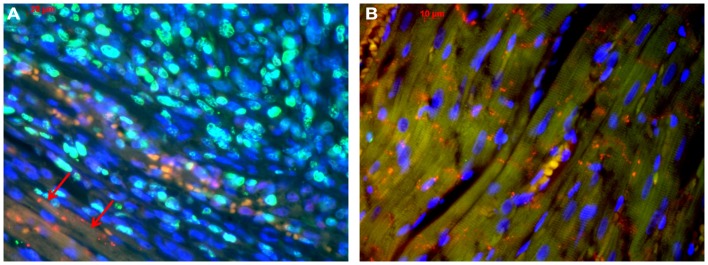
**Tumor-heart border [(A); red arrows mark the transition to the heart] and non-infiltrated normal heart tissue (B)**. Co-staining of Ki-67 (green), Cx43 (red), and nuclei (blue).

**Figure 6:**
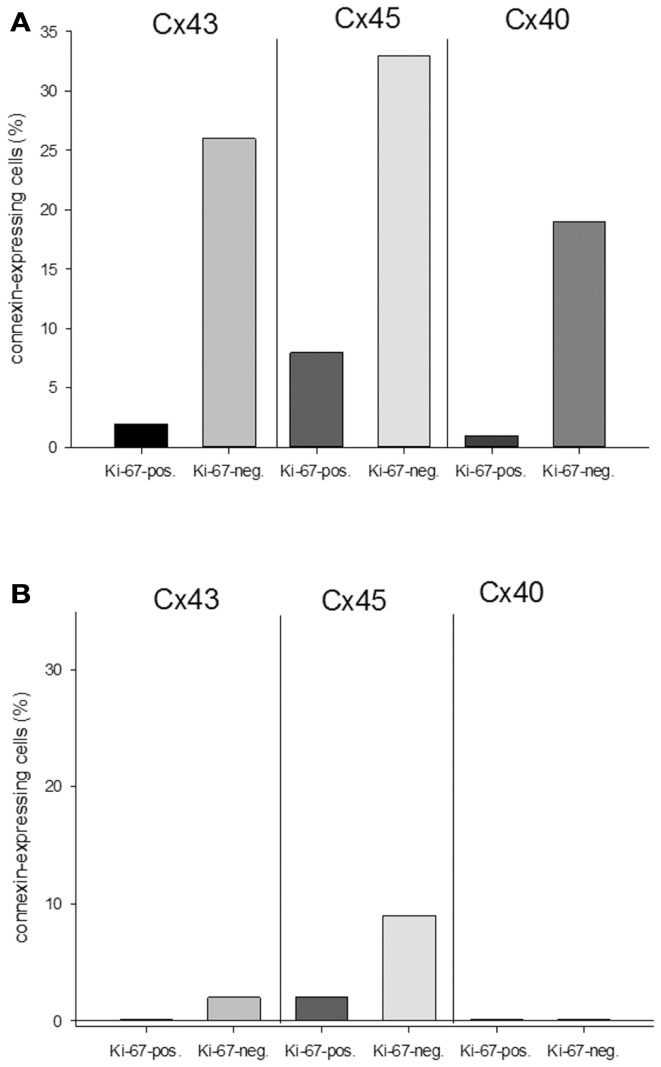
**Quantitative analysis of the relationship between connexin expression and the proliferation marker Ki-67 in the middle of the tumor (A) and at the tumor-heart border (B)**.

With regard to the question whether connexin expression and tumor growth may be interrelated we also investigated the expression of Ki-67, a marker of cell proliferation. In the middle of the tumor only 37 ± 2% of the cells were Ki-67 positive, while at the tumor-heart border significantly (*p* = 0.0007) more cells were Ki-67 positive, i.e., 51 ± 7% Ki-67^+^ cells. This was accompanied by a significant reduction in positivity for all cardiac connexins (*p* < 0.001) from 30 ± 6 to 2 ± 2% of the cells expressing any of the three connexins (*p* < 0.001). It became obvious, that cells which were positive for Ki-67 mostly were negative for Cx43, Cx40, or Cx45 (Figure 4). In particular, at the tumor-heart border, where the tumor infiltrated the heart, there were nearly no cells which showed immunopositivity for connexin (Figure 5). Quantitatively, in the middle of the tumor we found a clear statistical relationship between expression of Ki-67 and the lack connexin expression (Chi-square test: Cx43: *p* < 0.0001; Cx45: *p* < 0.03, Cx40: *p* < 0.014) where those cells expressing Ki-67 co-expressed significantly more rarely connexin (Figure 6A). At the tumor-heart border all cells expressing Ki-67 were negative for Cx43 and Cx40, and only 2% co-expressed Ki-67 and Cx45 (Figure 6B).

## Discussion

As a main finding we observed an inverse relationship between the expression of connexins, i.e., Cx43, Cx40, or Cx45, and of cell proliferation marker Ki-67 (which is not present in G0 phase). Moreover, in those parts of the tumor which invaded the heart, i.e., in the growth zone with highest Ki-67 expression, connexin expression was nearly absent. These data are in favor of the hypothesis that tumors cells are not communicating with their surrounding normal tissue when they grow invasively. In the present case the tumor did not express any of the typical cardiac connexins at the tumor-heart border where infiltrative growth was found. That could mean, that these tumor cells can not get growth-inhibitory signals from the normal cells *via* GJIC, thus, enabling invasive growth. This is similar to Mesnil et al. (1994) who found in a coculture of tumorigenic and non-tumorigenic rat liver epithelial cell lines that Cx43 was abundantly expressed in non-tumorigenic cells and was absent in tumorigenic cells. The zone lacking Cx43 indicated the boundary between tumorigenic and non-tumorigenic cells, similar to our tumor-heart border (Mesnil et al., 1994). The finding, that the sarcoma cells showed connexins rather predominantly intracytoplasmic than in the cytoplasmic membrane suggests that these connexins may not represent functional intercellular channels. A similar expression pattern has been shown for Cx32 and Cx43 in colorectal adenoma and even augmented in carcinoma (Kanczuga-Koda et al., 2010). By this aberrant localization, despite the expression of connexins in the tumor, the absence of detectable connexin signals in the membrane of the sarcoma cells probably means that they will not form functional gap junctions. This may lead to insufficient homologous GJIC within the tumor (Yamasaki et al., 1995). Similarly, Kalimi et al. (1992) reported of a reduction of homologous GJIC in v-raf- and v-raf/v-myc-transformed rat liver epithelial cells and a loss of heterologous GJIC in v-raf/v-myc-transformed cells by using scrape loading-dye transfer and fluorescence-recovery-after-photobleaching (FRAP) assays.

The role of GJIC in tumors is not entirely clear: on the one hand there are several tumors which do not express any connexin, e.g., HeLa cells (King et al., 2000a), and while many tumor promoting agents down regulate GJIC (Salameh and Dhein, 2005), and while tumor oncogenes like *ras, src, raf*, and *mos* have been shown to downregulate GJIC (Trosko et al., 2004). On the other hand there are also reports that suggest that connexins expressed in tumor cells may support the process of extravasation, invasion, and metastasis (Lin et al., 2002; Elzarrad et al., 2008). Regarding our study, in the tumor cells in our observation connexins are expressed. However, these connexins are obviously located predominantly if not solely intracellularly, so that they probably do not form functional channels. However, there was an interesting difference between the middle of the tumor and its invading border: while in the middle cells expressed connexins, they did not or almost not at the border.

The lack of membranous connexins would support the idea that the tumor cells do not communicate with the neighboring cells and, thus, can not receive growth inhibition signals from them. They are blind for the neighbor cells since they do not express the same connexins. Consequently, they do not see them and thus proliferate without inhibition. Although attractive, this view is probably too unilateral. Other signals may come from secreted growth inhibitors from other cells or from the microenvironment (Trosko et al., 1993; Barcellos-Hoff and Brooks, 2001). Regarding the role of connexins, we have to discriminate channel-related functions from channel-independent functions.

Thus, although not membrane-bound, connexin proteins are not functionless but can exert regulatory functions on growth and differentiation: it was shown that the carboxy terminal of Cx43 can associate with β-catenin. Thereby, Cx43 can regulate the cytosolic concentration of β-catenin. A reduction in Cx43, or phosphorylation at S262 by FGF-2, can increase the free cytosolic β-catenin which then in turn can translocate to the nucleus where it can activate TCF/LEF transcription factors and genes related to proliferation (like cyclin D, c-myc, c-jun) (Ai et al., 2000; Holnthoner et al., 2002; Doble and Woodgett, 2003). Furthermore the β-catenin-pool is regulated by wnt-Frizzled-receptor-signaling which reduces GSK3 activity. GSK3 constitutively phosphorylates β-catenin and thereby marks it for proteasomal degradation (Holnthoner et al., 2002; Doble and Woodgett, 2003).

Besides β-catenin, the secreted cysteine-rich and heparin binding protein CCN3 can interact with the carboxy terminal of Cx43 thereby inhibiting cellular proliferation (Fu et al., 2004; Gellhaus et al., 2004).

Among the abundant proteins which can interact with connexin43, zonula-occludens protein-1 (ZO-1), a scaffolding protein, is among the best investigated. The binding of ZO-1 to connexin43 also fixes its binding partner ZONAB (ZO-1 associated nucleic acid binding protein) together with CDK4 at the membrane and prevents from its translocation to the nucleus. If ZONAB is released it can together with CDK4 contribute to G1/S-transition (Giepmans and Moolenaar, 1998; Sourisseau et al., 2006). However, the binding of ZO-1 to connexin seems to be at least mostly related to membrane-bound connexin.

Taken together, besides acting as intercellular communication channel there is growing evidence that Cx43 can serve as an anti-proliferative nexus platform or binding “hub” for proteins which regulate growth. By docking to Cx43, the free concentration of these proteins is controlled. Depending on Cx43 concentration or phosphorylation status the other proteins may be released from the “hub” and translocate to the nucleus, where they can induce gene transcription linked to proliferation. Accordingly, a close relationship between Cx43 and important growth controlling genes was shown by Iacobas et al. (2005a,b).

This view is supported by our data showing that the increase in intracellular connexin is negatively related to the proliferation marker Ki-67.

Interestingly, the tumor data suggest that some factor in the middle of the tumor seemed to inhibit the synthesized connexins from being transported to the membrane. From the present data it remains unclear whether the connexin found intracellularly was monomeric or whether it was already oligomerized to connexons. Thus, it remains speculative to conclude on which level of the gap junction formation the process was arrested. However, it is tempting to speculate that an agent which would overcome this arrest may restore GJIC and thereby growth control.

Another issue worth some discussion is the stem cell. The observed sarcoma seemed to originate from the stem-cell-derived ET, which would mean that BM-MSC of a low passage (in that case sixth passage) were able to transform to a malignant tumor. While some investigators found functional connexins in MSC (Valiunas et al., 2004), other researchers reported that stem cells typically have no functional gap junctional intercellular communication and, thus, their growth control is assumed to be realized *via* secreted factors or other extracellular signals from their surrounding (Trosko et al., 2004). Accordingly, although the cells in our ET expressed connexins, these were predominantly located intracellularly. To become a tumor cell, it is necessary that a cell is immortalized and that it starts proliferation. Stem cells are considered to be immortal cells, which are under a non-gap-junction growth control, and according to the theory from Trosko et al. (2004) the carcinogenic process in this case would mean that this naturally immortal stem cell is prevented from mortalization or terminal differentiation. We can only speculate which factors in the present observation may have contributed, and besides scaffold factors it may be factors from the matrigel material or other factors yet unknown.

However, these observations finally may suggest that connexins like Cx43 might be interesting pharmacological anti-tumor targets. Drugs would need to re-establish the transfer of connexins to the membrane and their integration so that functional channels can be formed in those cells which express connexins intracellularly. Other drugs could act by re-expressing connexins and re-establishing GJIC. However, to achieve growth control this approach would require that the connexins re-expressed or re-integrated in the tumor are compatible with those of the surrounding normal tissue. This will not be possible in every case and may be problematic in particular for metastasis, since connexins may have a supportive role in the process of invasion/metastasis (Ito et al., 2000; Lin et al., 2002; Elzarrad et al., 2008). However, in a tumor as in the observed case, the proliferative invasive parts did not express connexins and re-establishing gap junctional intercellular communication could bear the chance to achieve growth control in these cells. There are some reports which show the principle feasibility of this approach (Zhang et al., 1998; King et al., 2000b; Momiyama et al., 2003).

### Limitations

As a limitation, it must be taken into account that we only tested for the three cardiac connexins, and not all 21 isoforms. However, since we were interested in the question whether the tumor may form gap junctions with the heart, these would have to contain the cardiac isoforms. Moreover, it would be interesting but was technically not possible to know if these tumor cells were definitively lacking any functional intercellular gap junction communication. Because of the use of a conventional fluorescence microscopy the optical section thickness could be variable. However, we used 4 μm thin sections.

Regarding the grading of the tumor and its entity, the previously described macroscopy of the tumor and the fact that the EHT consisted of 100% mesenchymal stem cells, led us to the assumption that it had to be a sarcoma originating from the MSC. The following histological examinations underlined this and suggested a pleomorphic sarcoma. However, since we did not investigate this by the use of detailed further immunohistochemistry, we cannot exclude the possibility, that it could also be a dedifferentiated high grade pleomorphic sarcoma (i.e., pleomorphic leiomyosarcoma, pleomorphic rhabdomyosarcoma) (Dei Tos, 2006). Moreover, it remains unclear whether the malignant transformation might be inborn to these cells or whether it might be caused/initiated by the treatment of these cells during the process of ET formation. However, this might be of less importance for the observation of lack of communication proteins at the tumor-heart border, which is the focus of the Present study.

## Conclusion

Taken together, we conclude that a pleomorphic sarcoma in the rat does not express connexins at the locations of invasive growth, and that proliferative activity and connexin expression are negatively correlated. Moreover, our data show that this type of tumor does not express tissue-specific connexins at the tumor tissue border. In consequence, we would underline the theory of Kalimi et al. (1992) that probably such a tumor does not get growth limitation signals from the surrounding normal tissue, which might lead to unlimited infiltrating growth. Regarding pharmacological perspectives a review of the current literature (see above) shows that re-establishing of GJIC may help to bring the cells under growth control again, but on the other hand it may also facilitate the metastatic process by enhancing the adhesivity of tumor cells to, e.g., vascular endothelium.

## Conflict of Interest Statement

The authors declare that the research was conducted in the absence of any commercial or financial relationships that could be construed as a potential conflict of interest.

## References

[B1] Ai Z. Fischer A. Spray D. C. Brown A. M. Fishman G. I. (2000). Wnt-1 regulation of connexin43 in cardiac myocytes. J. Clin. Invest. 105, 161–171 10.1172/JCI7798 10642594 PMC377428

[B2] Angervall L. Hagmar B. Kindblom L. G. Merck C. (1981). Malignant giant cell tumor of soft tissues: a clinicopathologic, cytologic, ultrastructural, angiographic, and microangiographic study. Cancer 47, 736–747 10.1002/1097-0142(19810215)47:4<736::AID-CNCR2820470419>3.0.CO;2-Q 7226022

[B3] Bang O. Y. Lee J. S. Lee P. H. Lee G. (2005). Autologous mesenchymal stem cell transplantation in stroke patients. Ann. Neurol. 57, 874–882 10.1002/ana.20501 15929052

[B4] Barcellos-Hoff M. H. Brooks A. L. (2001). Extracellular signaling through the microenvironment: a hypothesis relating carcinogenesis, bystander effects, and genomic instability. Radiat. Res. 156, 618–627 10.1667/0033-7587(2001)156[0618:ESTTMA]2.0.CO;2 11604083

[B5] Chin S. P. Poey A. C. Wong C. Y. Chang S. K. Teh W. Mohr T. J. (2010). Cryopreserved mesenchymal stromal cell treatment is safe and feasible for severe dilated ischemic cardiomyopathy. Cytotherapy 12, 31–37 10.3109/14653240903313966 19878080

[B6] Cronier L. Crespin S. Strale P. O. Defamie N. Mesnil M. (2009). Gap junctions and cancer: new functions for an old story. Antioxid. Redox Signal. 11, 323–338 10.1089/ars.2008.2153 18834328

[B7] Dei Tos A. P. (2006). Classification of pleomorphic sarcomas: where are we now? Histopathology 48, 51–62 10.1111/j.1365-2559.2005.02289.x 16359537

[B8] Deyrup A. T. Weiss S. W. (2006). Grading of soft tissue sarcomas: the challenge of providing precise information in an imprecise world. Histopathology 48, 42–50 10.1111/j.1365-2559.2005.02288.x 16359536

[B9] Djouad F. Plence P. Bony C. Tropel P. Apparailly F. Sany J. (2003). Immunosuppressive effect of mesenchymal stem cells favors tumor growth in allogeneic animals. Blood 102, 3837–3844 10.1182/blood-2003-04-1193 12881305

[B10] Doble B. W. Woodgett J. R. (2003). GSK-3: tricks of the trade for a multi-tasking kinase. J. Cell Sci. 116, 1175–1186 10.1242/jcs.00384 12615961 PMC3006448

[B11] Dobson K. R. Reading L. Haberey M. Marine X. Scutt A. (1999). Centrifugal isolation of bone marrow from bone: an improved method for the recovery and quantitation of bone marrow osteoprogenitor cells from rat tibiae and femurae. Calcif. Tissue Int. 65, 411–413 10.1007/s002239900723 10541770

[B12] Elzarrad M. K. Haroon A. Willecke K. Dobrowolski R. Gillespie Elzarrad, M. K. Haroon A. Willecke K. Dobrowolski R. Gillespie M. N. Al-Mehdi A. B. (2008). Connexin-43 upregulation in micrometastases and tumor vasculature and its role in tumor cell attachment to pulmonary endothelium. BMC Med. 6:20 10.1186/1741-7015-6-20 18647409 PMC2492868

[B13] Evans W. H. Martin P. (2002). Gap junctions: structure and function. Mol. Membr. Biol. 19, 121–136 10.1080/09687680210139839 12126230

[B14] Ezumi K. Yamamoto H. Murata K. Higashiyama M. Damdinsuren B. Nakamura Y. (2008). Aberrant expression of connexin 26 is associated with lung metastasis of colorectal cancer. Clin. Cancer Res. 14, 677–684 10.1158/1078-0432.CCR-07-1184 18245526

[B15] Fletcher C. D. (1992). Pleomorphic malignant fibrous histiocytoma: fact or fiction? A critical reappraisal based on 159 tumors diagnosed as pleomorphic sarcoma. Am. J. Surg. Pathol. 16, 213–228 10.1097/00000478-199204000-00006 1317996

[B16] Fu C. T. Bechberger J. F. Ozog M. A. Perbal B. Naus C. C. (2004). CCN3 (NOV) interacts with connexin43 in C6 glioma cells: possible mechanism of connexin-mediated growth suppression. J. Biol. Chem. 279, 36943–36950 10.1074/jbc.M311061200 15213231

[B17] Gellhaus A. Dong X. Propson S. Maass K. Klein-Hitpass L. Kibschull M. (2004). Connexin43 interacts with NOV: a possible mechanism for negative regulation of cell growth in choriocarcinoma cells. J. Biol. Chem. 279, 36931–36942 10.1074/jbc.M404073200 15181016

[B18] Giepmans B. N. Moolenaar W. H. (1998). The gap junction protein connexin43 interacts with the second PDZ domain of the zona occludens-1 protein. Curr. Biol. 8, 931–934 10.1016/S0960-9822(98)00013-X 9707407

[B19] Guccion J. G. Enzinger F. M. (1972). Malignant giant cell tumor of soft parts. An analysis of 32 cases. Cancer 29, 1518–1529 10.1002/1097-0142(197206)29:6<1518::AID-CNCR2820290616>3.0.CO;2-# 5031245

[B20] Guillou L. Coindre J. M. Bonichon F. Nguyen B. B. Terrier P. Collin F. (1997). Comparative study of the National Cancer Institute and French Federation of Cancer Centers Sarcoma Group grading systems in a population of 410 adult patients with soft tissue sarcoma. J. Clin. Oncol. 15, 350–362 8996162 10.1200/JCO.1997.15.1.350

[B21] Gunn W. G. Conley A. Deininger L. Olson S. D. Prockop D. J. Gregory C. A. (2006). A crosstalk between myeloma cells and marrow stromal cells stimulates production of DKK1 and interleukin-6: a potential role in the development of lytic bone disease and tumor progression in multiple myeloma. Stem Cells 24, 986–991 10.1634/stemcells.2005-0220 16293576

[B22] Hagen A. Dietze A. Dhein S. (2009). Human cardiac gap junction coupling: effects of antiarrhythmic peptide AAP10. Cardiovasc. Res. 83, 405–415 10.1093/cvr/cvp028 19176598

[B23] Holnthoner W. Pillinger M. Groger M. Wolff K. Ashton A. W. Albanese C. (2002). Fibroblast growth factor-2 induces Lef/Tcf-dependent transcription in human endothelial cells. J. Biol. Chem. 277, 45847–45853 10.1074/jbc.M209354200 12235165

[B24] Hong D. Chen H. X. Xue Y. Li D. M. Wan X. C. Ge R. (2009). Osteoblastogenic effects of dexamethasone through upregulation of TAZ expression in rat mesenchymal stem cells. J. Steroid Biochem. Mol. Biol. 116, 86–92 10.1016/j.jsbmb.2009.05.007 19460432

[B25] Horwitz E. M. Prockop D. J. Fitzpatrick L. A. Koo W. W. Gordon P. L. Neel M. (1999). Transplantability and therapeutic effects of bone marrow-derived mesenchymal cells in children with osteogenesis imperfecta. Nat. Med. 5, 309–313 10.1038/6529 10086387

[B26] Iacobas D. A. Iacobas S. Li W. E. Zoidl G. Dermietzel R. Spray D. C. (2005a). Genes controlling multiple functional pathways are transcriptionally regulated in connexin43 null mouse heart. Physiol. Genomics 20, 211–223 15585606 10.1152/physiolgenomics.00229.2003

[B27] Iacobas D. A. Iacobas S. Spray D. C. (2005b). “Use of cDNA aarays to explore gene expression in genetically manipulated mice and cell lines,” in Practical Methods in Cardiovascular Research, eds Dhein S. Mohr F. W. Delmar M. (Berlin: Springer), 907–915

[B28] Ito A. Katoh F. Kataoka T. R. Okada M. Tsubota N. Asada H. (2000). A role for heterologous gap junctions between melanoma and endothelial cells in metastasis. J. Clin. Invest. 105, 1189–1197 10.1172/JCI8257 10791993 PMC315440

[B29] Kalimi G. H. Hampton L. L. Trosko J. E. Thorgeirsson S. S. Huggett A. C. (1992). Homologous and heterologous gap-junctional intercellular communication in v-raf-, v-myc-, and v-raf/v-myc-transduced rat liver epithelial cell lines. Mol. Carcinog. 5, 301–310 10.1002/mc.2940050411 1379816

[B30] Kanczuga-Koda L. Koda M. Sulkowski S. Wincewicz A. Zalewski B. Sulkowska M. (2010). Gradual loss of functional gap junction within progression of colorectal cancer – a shift from membranous CX32 and CX43 expression to cytoplasmic pattern during colorectal carcinogenesis. In vivo 24, 101–107 20133984

[B31] Karnoub A. E. Dash A. B. Vo A. P. Sullivan A. Brooks M. W. Bell G. W. (2007). Mesenchymal stem cells within tumour stroma promote breast cancer metastasis. Nature 449, 557–563 10.1038/nature06188 17914389

[B32] Karussis D. Kassis I. Kurkalli B. G. Slavin S. (2008). Immunomodulation and neuroprotection with mesenchymal bone marrow stem cells (MSCs): a proposed treatment for multiple sclerosis and other neuroimmunological/neurodegenerative diseases. J. Neurol. Sci. 265, 131–135 10.1016/j.jns.2007.05.005 17610906

[B33] Khakoo A. Y. Pati S. Anderson S. A. Reid W. Elshal M. F. Rovira I. I. (2006). Human mesenchymal stem cells exert potent antitumorigenic effects in a model of Kaposi’s sarcoma. J. Exp. Med. 203, 1235–1247 10.1084/jem.20051921 16636132 PMC2121206

[B34] King T. J. Fukushima L. H. Donlon T. A. Hieber A. D. Shimabukuro K. A. Bertram J. S. (2000a). Correlation between growth control, neoplastic potential and endogenous connexin43 expression in HeLa cell lines: implications for tumor progression. Carcinogenesis 21, 311–315 10.1093/carcin/21.2.311 10657974

[B35] King T. J. Fukushima L. H. Hieber A. D. Shimabukuro K. A. Sakr W. A. Bertram J. S. (2000b). Reduced levels of connexin43 in cervical dysplasia: inducible expression in a cervical carcinoma cell line decreases neoplastic potential with implications for tumor progression. Carcinogenesis 21, 1097–1109 10.1093/carcin/21.2.311 10836996

[B36] Kurtova A. V. Balakrishnan K. Chen R. Ding W. Schnabl S. Quiroga M. P. (2009). Diverse marrow stromal cells protect CLL cells from spontaneous and drug-induced apoptosis: development of a reliable and reproducible system to assess stromal cell adhesion-mediated drug resistance. Blood 114, 4441–4450 10.1182/blood-2009-07-233718 19762485 PMC4081374

[B37] Laird D. W. Fistouris P. Batist G. Alpert L. Huynh H. T. Carystinos G. D. (1999). Deficiency of connexin43 gap junctions is an independent marker for breast tumors. Cancer Res. 59, 4104–4110 10463615

[B38] Lampe P. D. (1994). Analyzing phorbol ester effects on gap junctional communication: a dramatic inhibition of assembly. J. Cell Biol. 127, 1895–1905 10.1083/jcb.127.6.1895 7806568 PMC2120282

[B39] Leontyev S. Schlegel F. Spath C. Schmiedel R. Nichtitz M. Boldt A. (2013). Transplantation of engineered heart tissue (EHT) as biological cardiac assist device for treatment of dilated cardiomyopathy. Eur. J. Heart Fail. 15, 23–35 10.1093/eurjhf/hfs200 23243122

[B40] Lin J. H. Takano T. Cotrina M. L. Arcuino G. Kang J. Liu S. (2002). Connexin43 enhances the adhesivity and mediates the invasion of malignant glioma cells. J. Neurosci. 22, 4302–4311 12040035 10.1523/JNEUROSCI.22-11-04302.2002 PMC6758793

[B41] Loewenstein W. R. (1980). Junctional cell-to-cell communication and growth control. Ann. N. Y. Acad. Sci. 339, 39–45 10.1111/j.1749-6632.1980.tb15966.x 6249141

[B42] Loewenstein W. R. Kanno Y. (1966). Intercellular communication and the control of tissue growth: lack of communication between cancer cells. Nature 209, 1248–1249 10.1038/2091248a0 5956321

[B43] Maestroni G. J. Hertens E. Galli P. (1999). Factor(s) from nonmacrophage bone marrow stromal cells inhibit Lewis lung carcinoma and B16 melanoma growth in mice. Cell. Mol. Life Sci. 55, 663–667 10.1007/s000180050322 10357234 PMC11147120

[B44] Mesnil M. Asamoto M. Piccoli C. Yamasaki H. (1994). Possible molecular mechanism of loss of homologous and heterologous gap junctional intercellular communication in rat liver epithelial cell lines. Cell Adhes. Commun. 2, 377–384 10.3109/15419069409004449 7842253

[B45] Mesnil M. Crespin S. Avanzo J. L. Zaidan-Dagli M. L. (2005). Defective gap junctional intercellular communication in the carcinogenic process. Biochim. Biophys. Acta 1719, 125–145 10.1016/j.bbamem.2005.11.004 16359943

[B46] Miura M. Miura Y. Padilla-Nash H. M. Molinolo A. A. Fu B. Patel V. (2006). Accumulated chromosomal instability in murine bone marrow mesenchymal stem cells leads to malignant transformation. Stem Cells 24, 1095–1103 10.1634/stemcells.2005-0403 16282438

[B47] Momiyama M. Omori Y. Ishizaki Y. Nishikawa Y. Tokairin T. Ogawa J. (2003). Connexin26-mediated gap junctional communication reverses the malignant phenotype of MCF-7 breast cancer cells. Cancer Sci. 94, 501–507 10.1111/j.1349-7006.2003.tb01473.x 12824874 PMC11160258

[B48] Nagaya N. Kangawa K. Itoh T. Iwase T. Murakami S. Miyahara Y. (2005). Transplantation of mesenchymal stem cells improves cardiac function in a rat model of dilated cardiomyopathy. Circulation 112, 1128–1135 10.1161/CIRCULATIONAHA.104.500447 16103243

[B49] Nakamura K. Ito Y. Kawano Y. Kurozumi K. Kobune M. Tsuda H. (2004). Antitumor effect of genetically engineered mesenchymal stem cells in a rat glioma model. Gene Ther. 11, 1155–1164 10.1038/sj.gt.3302228 15141157

[B50] Naoi Y. Miyoshi Y. Taguchi T. Kim S. J. Arai T. Tamaki Y. (2007). Connexin26 expression is associated with lymphatic vessel invasion and poor prognosis in human breast cancer. Breast Cancer Res. Treat. 106, 11–17 10.1007/s10549-006-9465-8 17203385

[B51] Naus C. C. Laird D. W. (2010). Implications and challenges of connexin connections to cancer. Nat. Rev. Cancer 10, 435–441 10.1038/nrc2841 20495577

[B52] Ohlsson L. B. Varas L. Kjellman C. Edvardsen K. Lindvall M. (2003). Mesenchymal progenitor cell-mediated inhibition of tumor growth in vivo and in vitro in gelatin matrix. Exp. Mol. Pathol. 75, 248–255 10.1016/j.yexmp.2003.06.001 14611816

[B53] Pittenger M. F. Mackay A. M. Beck S. C. Jaiswal R. K. Douglas R. Mosca J. D. (1999). Multilineage potential of adult human mesenchymal stem cells. Science 284, 143–147 10.1126/science.284.5411.143 10102814

[B54] Romanov Y. A. Svintsitskaya V. A. Smirnov V. N. (2003). Searching for alternative sources of postnatal human mesenchymal stem cells: candidate MSC-like cells from umbilical cord. Stem Cells 21, 105–110 10.1634/stemcells.21-1-105 12529557

[B55] Rubio D. Garcia-Castro J. Martín M. C. de la Fuente R. Cigudosa J. C. Lloyd A. C. (2005). Spontaneous human adult stem cell transformation. Cancer Res. 65, 3035–3039 15833829 10.1158/0008-5472.CAN-04-4194

[B56] Saito-Katsuragi M. Asada H. Niizeki H. Katoh F. Masuzawa M. Tsutsumi M. (2007). Role for connexin 26 in metastasis of human malignant melanoma: communication between melanoma and endothelial cells via connexin 26. Cancer 110, 1162–1172 10.1002/cncr.22894 17614106

[B57] Salameh A. Dhein S. (2005). Pharmacology of gap junctions. New pharmacological targets for treatment of arrhythmia, seizure and cancer? Biochim. Biophys. Acta 1719, 36–58 10.1016/j.bbamem.2005.09.007 16216217

[B58] Sato H. Hagiwara H. Senba H. Fukumoto K. Nagashima Y. Yamasaki H. (2008). The inhibitory effect of connexin 32 gene on metastasis in renal cell carcinoma. Mol. Carcinog. 47, 403–409 10.1002/mc.20396 18058801

[B59] Söhl G. Willecke K. (2004). Gap junctions and the connexin protein family. Cardiovasc. Res. 62, 228–232 10.1016/j.cardiores.2003.11.013 15094343

[B60] Sourisseau T. Georgiadis A. Tsapara A. Ali R. R. Pestell R. Matter K. (2006). Regulation of PCNA and cyclin D1 expression and epithelial morphogenesis by the ZO-1-regulated transcription factor ZONAB/DbpA. Mol. Cell. Biol. 26, 2387–2398 10.1128/MCB.26.6.2387-2398.2006 16508013 PMC1430269

[B61] Spaeth E. L. Dembinski J. L. Sasser A. K. Watson K. Klopp A. Hall B. (2009). Mesenchymal stem cell transition to tumor-associated fibroblasts contributes to fibrovascular network expansion and tumor progression. PLoS ONE 4:e4992 10.1371/journal.pone.0004992 19352430 PMC2661372

[B62] Strawn W. B. Richmond R. S. Ann Tallant E. Gallagher P. E. Ferrario C. M. (2004). Renin-angiotensin system expression in rat bone marrow haematopoietic and stromal cells. Br. J. Haematol. 126, 120–126 10.1111/j.1365-2141.2004.04998.x 15198742

[B63] Tang J. Wang J. Yang J. Kong X. Zheng F. Guo L. (2009). Mesenchymal stem cells over-expressing SDF-1 promote angiogenesis and improve heart function in experimental myocardial infarction in rats. Eur. J. Cardiothorac. Surg. 36, 644–650 10.1016/j.ejcts.2009.04.052 19524448

[B64] Tian K. Yang S. Ren Q. Han Z. Lu S. Ma F. (2010). p38 MAPK contributes to the growth inhibition of leukemic tumor cells mediated by human umbilical cord mesenchymal stem cells. Cell. Physiol. Biochem. 26, 799–808 10.1159/000323973 21220911

[B65] Tolar J. Nauta A. J. Osborn M. J. Panoskaltsis Mortari A. McElmurry R. T. Bell S. (2007). Sarcoma derived from cultured mesenchymal stem cells. Stem Cells 25, 371–379 10.1634/stemcells.2005-0620 17038675

[B66] Trosko J. E. Chang C. C. Madhukar B. V. Dupont E. (1993). “Oncogenesis, tumor suppressor genes, and intercellular communication in the ‘oncogeny as partially blocked ontogeny’ hypothesis,” in New Frontiers in Cancer Causation, ed. Iversen O. H. (Washington: Taylor & Francis), 181–197

[B67] Trosko J. E. Chang C. C. Madhukar B. V. Klaunig J. E. (1990). Chemical, oncogene and growth factor inhibition gap junctional intercellular communication: an integrative hypothesis of carcinogenesis. Pathobiology 58, 265–278 10.1159/000163596 2076190

[B68] Trosko J. E. Chang C. C. Upham B. L. Tai M. H. (2004). Ignored hallmarks of carcinogenesis: stem cells and cell-cell communication. Ann. N. Y. Acad. Sci. 1028, 192–201 10.1196/annals.1322.023 15650245

[B69] Valiunas V. Doronin S. Valiuniene L. Potapova I. Zuckerman J. Walcott B. (2004). Human mesenchymal stem cells make cardiac connexins and form functional gap junctions. J. Physiol. (Lond.) 555(Pt 3), 617–626 10.1113/jphysiol.2003.058719 14766937 PMC1664864

[B70] Wan X. C. Liu C. P. Li M. Hong D. Li D. M. Chen H. X. (2008). Staphylococcal enterotoxin C injection in combination with ascorbic acid promotes the differentiation of bone marrow-derived mesenchymal stem cells into osteoblasts in vitro. Biochem. Biophys. Res. Commun. 373, 488–492 10.1016/j.bbrc.2008.06.037 18572015

[B71] Wong R. S. (2011). Mesenchymal stem cells: angels or demons? J. Biomed. Biotechnol. 2011, 459510 10.1155/2011/413802 21822372 PMC3142786

[B72] Yamasaki H. Mesnil M. Omori Y. Mironov N. Krutovskikh V. (1995). Intercellular communication and carcinogenesis. Mutat. Res. 333, 181–188 10.1016/0027-5107(95)00144-1 8538626

[B73] Yano T. Fujimoto E. Hagiwara H. Sato H. Yamasaki H. Negishi E. (2006). Connexin 32 as an anti-invasive and anti-metastatic gene in renal cell carcinoma. Biol. Pharm. Bull. 29, 1991–1994 10.1248/bpb.29.1006 17015938

[B74] Zhang Z. Q. Zhang W. Wang N. Q. Bani-Yaghoub M. Lin Z. X. Naus C. C. (1998). Suppression of tumorigenicity of human lung carcinoma cells after transfection with connexin43. Carcinogenesis 19, 1889–1894 10.1093/carcin/19.11.1889 9854998

[B75] Zhou Y. F. Bosch-Marce M. Okuyama H. Krishnamachary B. Kimura H. Zhang L. (2006). Spontaneous transformation of cultured mouse bone marrow-derived stromal cells. Cancer Res. 66, 10849–10854 10.1158/0008-5472.CAN-06-1726 17108121

[B76] Zhu W. Xu W. Jiang R. Qian H. Chen M. Hu J. (2006). Mesenchymal stem cells derived from bone marrow favor tumor cell growth in vivo. Exp. Mol. Pathol. 80, 267–274 10.1016/j.yexmp.2005.07.004 16214129

[B77] Zimmermann W. H. Melnychenko I. Wasmeier G. Didie M. Naito H. Nixdorff U. (2006). Engineered heart tissue grafts improve systolic and diastolic function in infarcted rat hearts. Nat. Med. 12, 452–458 10.1038/nm1394 16582915

[B78] Zuk P. A. Zhu M. Mizuno H. Huang J. Futrell J. W. Katz A. J. (2001). Multilineage cells from human adipose tissue: implications for cell-based therapies. Tissue Eng. 7, 211–228 10.1089/107632701300062859 11304456

